# Successful Dental Operations

**Published:** 1860-06

**Authors:** 


### SUCCESSFUL DENTAL OPERATIONS.

In the exact ratio of soul agreement between the operator and patient will success crown their coming in contact.

*Temperament* and tastes alike—both are more likely to be pleased with each other, which is one of the prime conditions of fitness to operate or be operated upon. Let antagonism spring up between an operator and his patient and my point will soon be proved, for they will be in haste to get rid of each other, and for the time give more thought to this *feeling* than to a cool and sensible calculation of how much they can combine their efforts, earnestly to attain the highest results for both.

Careless, rough, *unfeeling* operators, should never have patients upon whom to display these repulsive qualities, no more than testy, squamish, unreasonable, exacting patients should demand only the delicate, feeling, competent, and good operator to serve them; not only *doing* the *work* in the best possible manner, but, also, required to bear their part and the patients also, unduly wearing themselves away for unappreciated services. For all such incongruity tends to the performance of a service far from successful, in the sense satisfactory to the after thought of either conscientious operator or honest patient.

The anxiety to get through an irksome task often proves an efficient barrier in the way of success—resulting in getting what they call "through-done." But be assured all such operations are not permanent or paramount.

No operation should be "irksome" to patient or operator in any case, and I am happy to be enabled to announce "the time has come when there is no longer any *need* that it should thus be."

Any operator who attempts to operate when he is not in proper condition is sinning against not only himself, but his profession, as well as those he essays to serve under such circumstances, as certainly as if he attempted to exercise his handicraft, (I will not say skill) on an organ, prematurely! because he lacked knowledge, time, patience or honesty to first put it in proper condition to ensure a certainty of restoration.

When I say that it is necessary to success, that an operator come near the "heart" (feelings) of his patient (if fine) I do not mean to endorse in the slightest degree that mawkish palaver and senseless expression of regard, that is the best evidence of absence of real interest in the personality in question, in every sense but the low one of what *money* can be made by such "*arts.*" But I do wish to be understood to affirm that it is necessary for the highest rendering of dental service, that we make every step, from the first inception of the survey of the case all the way through, a careful and lucid diagnosis and completion of the necessary work, a matter of personal religious duty. To do which there is usually but one special barrier, out of the range of the operator's own mind, viz: that unfortunate element called price. If the dentist could be so situated as to take no thought of this at all until after all the elements of price had been made to enter into the work, a much more satisfactory, because more strictly intelligent mode of arriving at this vexed question would have been secured. The only successful operations are those that are absolutely *thorough*, no half-way "guess it will do" sort of work *can* fulfill the demand for the only kind of work that should ever be suffered to be put into the mouth of a human being!

For be it kept in mind that the moral state is in a great degree dependent upon physical conditions, especially of the digestive apparatus. How many times have we been almost entrapped into this loose way of turning off patients for fear they would think it was for the pay in the pecuniary sense, we were so sharp in finding all the incipient cavities and pointing out the advantage of an early attention to them!

I presume every operator of considerable experience has at some time in his life had cause to regret his cowardice, lack of manly independence in just such cases, when his patient has returned to have a tooth "*extracted*," that had but a small cavity in it, when last in the chair. And had been notified to have an eye upon that "small spot" that has alas! proved to have been far above the estimate put upon its size and danger—now calling for the exercise of a skill probably out of his or his patient's reach! Out of his when he does know what to do—out of his patient's when the enhanced expense of time and money have gone beyond his ability to meet. And so to get rid of a monitor of the ignorance or neglect of other days, quacklike, closes the tragedy by a "coup de-main," and buries out of sight the patent evidence of his weakness—imploring the ability to be able to forget it "that it may be no more a grievous remembrance to him," instead of nailing it in a sure place, that it might be a beacon light enabling him "no more to stand on that particular coast." Thus we have the difficulties, out of ourselves, narrowed down to a single element—Price. For please make particular note, no one will find fault with your services because of their preëminent excellence, if that same bug-bear, *price*, be no part the preëminence. I never have offended any one, when doing gratuitous service on the ground of doing it too well.

Though, nevertheless, some of this class of service has cost me most of time, money, and earnest effort, and I must say has paid my *feelings* better than my highest and most happy successes, where I received pecuniary reward, because above the suspicion of being done for money!

How shall this last *outside* foe be slain?

I. By preparing ourselves to do a worthy, honorable service, without reference to the things to be added—to follow.

II. By convincing the public of the residence of this paramount necessity, in us as a profession.

III. By actualizing our pretentions in careful and correct examinations of *all* the cases with a view to *know* that we may *do* the highest possible service the special example in hand admits. The first step of which is to study the *temperament*—that we may know how to govern ourselves toward the patient, and what to expect of the patient towards us. After we are satisfied one way or the other. If we are incompatibles, kindly, but firmly and frankly tell the patient so and honorably dismiss him in justice to him, as well as to ourselves; for there may be a good operator who is compatible to him and he thereby secure a *successful* service. If esteemed compatibles, the next step will be to *certainly* diagnose the case, upon which either favorable or unfavorable prognosis necessarily depends. If it is deemed proper to proceed to do the work, the way is now made open for a successful operation. If not thought best by one or both to proceed, your *labor* is at an end for which, in justice to all concerned, you should demand and receive a proper compensation, the amount of which no one can so well determine as yourself. In case you proceed to perform an operation—patient, thorough, self-sacrificing effort on your part, will secure the desired end, just in the degree that you first conceive and then fulfill these varied requests. Our victory being now complete as against outside foes and our competency also established, a true professional standing will have been attained.

Does any one require a definition of the true Professional Man?

Not only a gentleman, a scholar, and expert in the use of tools, (I suppose I must say by way of deference instruments) but also a truly religious benevolent man!

Some no doubt would insert christian, well, I would too if not for the multifarious significations that name presents to the perception, many of which did not find soul-room in the blessed Nazarene.

There is a feeble sketch of my scientific successful Dentist. S.

